# Root Branching and Nutrient Efficiency: Status and Way Forward in Root and Tuber Crops

**DOI:** 10.3389/fpls.2019.00237

**Published:** 2019-03-04

**Authors:** Luis O. Duque, Arthur Villordon

**Affiliations:** ^1^Department of Plant Science, The Pennsylvania State University, University Park, PA, United States; ^2^Sweet Potato Research Station, Louisiana State University Agricultural Center, Chase, LA, United States

**Keywords:** root system architecture (RSA), root and tuber crops, nutrient efficiency, sweetpotato, potato, yam, cassava

## Abstract

Plants are immobile organisms that require roots to efficiently and cost-effectively exploit their habitat for water and nutrients. Plant root systems are dynamic structures capable of altering root branching, root angle, and root growth rates determining overall architecture. This plasticity involves belowground plant-root mediated synergies coupled through a continuum of environmental interactions and endogenous developmental processes facilitating plants to adapt to favorable or adverse soil conditions. Plant root branching is paramount to ensure adequate access to soil water and nutrients. Although substantial resources have been devoted toward this goal, significant knowledge gaps exist. In well-studied systems such as rice and maize, it has become evident that root branching plays a significant role in the acquisition of nutrients and other soil-based resources. In these crop species, specific root branching traits that confer enhanced nutrient acquisition are well-characterized and are already being incorporated into breeding populations. In contrast, the understanding of root branching in root and tuber crop productivity has lagged behind. In this review article, we highlight what is known about root branching in root and tuber crops (RTCs) and mark new research directions, such as the use novel phenotyping methods, examining the changes in root morphology and anatomy under nutrient stress, and germplasm screening with enhanced root architecture for more efficient nutrient capture. These directions will permit a better understanding of the interaction between root branching and nutrient acquisition in these globally important crop species.

## Introduction

A plant’s ability to explore the soil and to compete for soil resources is largely dependent on the architecture of its root system (Lynch, 1995). Root system architecture (RSA) is determined by the pattern of root branching and by the rate and trajectory of growth of individual roots (Zhang et al., 1999). There is scientific consensus that root branching is subject to genetic control and influenced by biotic and abiotic factors. Therefore, manipulating RSA has emerged as a fundamental strategy to enhance nutrient acquisition especially in low input agricultural systems. For example, Gamuyao et al. (2012) documented the presence of a Pup1-specific protein kinase gene, the phosphorus-starvation tolerance 1 (PSTOL1) derived from the traditional aus-type rice variety Kasalath. This protein kinase was shown to enhance early root growth, thereby enabling plants to acquire more phosphorus and other nutrients under phosphorus-deficient soils. Gamuyao et al. (2012) suggested that introduction of this quantitative trait locus into locally adapted rice cultivars in Asia and Africa could enhance productivity under low nutrient conditions. Follow-up work by Neelam et al. (2017) documents novel alleles of *PSTOL1* in *Oryza rufipogon*, the Asian wild rice, and work is already ongoing to introduce these alleles in elite rice cultivars. This and other similar examples underscore the importance of gaining a comprehensive understanding of root architecture adaptations that could contribute to productivity under marginal or low-input growing conditions. Low soil fertility in developing countries is a primary constraint to food security and economic development (Rao et al., 2016). In Africa in particular, depletion of soil fertility is a major biophysical cause of low per capita food production, contributing to food insecurity in the region (Sanchez, 2002). Increasing the capacity of plants to acquire soil resources is a key approach to improve crop yields and reduce farmer’s dependence on fertilizers (Bishopp and Lynch, 2015). The cereal species wheat and rice provide more than 50% of the calories consumed by humans (Rich and Watt, 2013). However, root and tuber crops (RTCs) are second in importance to cereals as a global source of carbohydrates, and grown in regions not suitable for cereal production^1^. In this work, we focus mainly on cassava (*Manihot esculenta*), potato (*Solanum tuberosum*), sweetpotato (*Ipomoea batatas*) and yams (*Dioscorea sp*), which the Food and Agriculture Organization defines as among the primary root and tuber crops of global importance^2^.

## Root Architecture and Nutrient Efficiency in Root and Tuber Crops: The Current State of Knowledge

Two comprehensive reviews of literature regarding root architecture in RTCs were conducted in 2014 and 2016 (Villordon et al., 2014; Khan et al., 2016). Curiously enough, in a comprehensive review of the subject matter, it was determined that between 2004 and 2014, there was only one published work on the subject of root morphological description for each of the RTCs compared to 12 for maize (Villordon et al., 2014). In the current work, we surveyed articles published within the past 10 years that specifically address the relationship of root architecture in response to heterogenous nutrient environments in RTCs (Table 1).

**Table 1:** Survey of articles published within the past 10 years that address root architecture and NPK acquisition in rice, cassava, sweetpotato, potato, and yams.

Nutrient	Crop species	Reference
Nitrogen	Rice	Obara et al., 2010; Ji et al., 2012; Ogawa et al., 2014; Ju et al., 2015; Kim et al., 2015; Selvaraj et al., 2017
	Sweetpotato	Villordon et al., 2013, 2014
Phosphorus	Rice	Shimizu et al., 2008; Fang et al., 2009; Li et al., 2009; Dai et al., 2012; Gamuyao et al., 2012; Shen et al., 2013; Takehisa et al., 2013; Topp et al., 2013; Vejchasarn et al., 2016
	Potato	Balemi and Schenk, 2009; Wang et al., 2015; Krell et al., 2018; White et al., 2018
	Sweetpotato	Villordon et al., 2018
Potassium	Rice	Jia et al., 2008; Ma et al., 2012


Crops frequently alter both their aboveground as well as their belowground structures morphologically and physiologically in response to heterogeneous nutrient environments (Drew, 1975; Fransen et al., 1999; Hodge, 2004), in which yield and nutrient uptake capabilities surpass those in nutrient-homogeneous environments. Of these soil mineral nutrients, nitrogen (N), phosphorus (P) and potassium (K) are considered the most important for crop growth, development and subsequent yield. However, phytoavailability of NPK often limits low-input agriculture (Mueller et al., 2012; White et al., 2013). For comparison, we included references available for the relationship between root architecture manipulation and NPK uptake in rice. The survey reveals a disturbing trend: the number of publications on rice exceeds the combined scientific output of the RTCs. Relative to the rice knowledge base, there is a substantial knowledge gap in RTCs about the role of RSA in the exploration and acquisition of nutrients in low-input environments. The survey reveals that rice RSA research has focused on N and P. This is consistent with current understanding that N and P availability are the primary global constraints and particularly severe in low-input agriculture characteristic of many developing nations (Lynch, 2007). N compounds are mobile and prone to leaching into deeper soils. In contrast, P accumulates mainly in the topsoil in part due to its low mobility. Among the RTCs, potato leads in terms of research output, primarily focusing on P. This is in part due to the large P requirement in the crop, about two-fold higher compared to that of cereal crops such as wheat and barley and 1/3 higher compared to most vegetable crops (White et al., 2018). The published studies on potato RSA and P, although relatively fewer compared to rice, has led to direct applications in terms of identifying desirable root traits for improved P acquisition and the identification of cultivars and genotypes with improved P efficiency in low nutrient conditions (White et al., 2005, 2018; Wang et al., 2015; Krell et al., 2018).

In sweetpotato, the published reports on N and P represent translational research of key findings from model systems (i.e., *Arabidopsis*, maize and rice). First, Villordon et al. (2013) demonstrated that lateral root branching jointly measured as lateral root length, number of lateral roots and lateral root density in sweetpotato cv. Beauregard was altered in response to variation in overall available N. The variation in RSA in response to different available N was consistent with prior work in *Arabidopsis* where external N presence had stimulatory effect on lateral root elongation, whereas high N concentrations inhibited lateral root meristem activity (Zhang et al., 2007). The data regarding relationship between spatial N availability and RSA modifications were similar to findings in *Arabidopsis* model systems that localized N availability is critical for lateral root signaling and development (Zhang and Forde, 2000; Zhang et al., 2007; Lima et al., 2010). Second, Villordon et al. (2018) reported that storage root length in sweetpotato cvs. Bayou Belle and Beauregard varied in response to experimental P deficiency. These findings corroborate available experimental evidence in *Arabidopsis* model systems that support the hypothesis that the root tip is the site of P sensing and that optimal or low P is involved in the growth or arrest of primary root growth (Svistoonoff et al., 2007; Kellermeier et al., 2014; Medici et al., 2015; Abel, 2017).

With regards to K, Liu et al. (2017) showed differences in root morphology under controlled K and deficient K treatments in two representative sweetpotato cultivars, Ningzishu 1 (sensitive to K deficiency) and Xushu 32 (tolerant to K deficiency). Under K deficiency, root length, surface area, root volume and average root diameter was reduced in Ningzishu 1 compared to Xushu 32. Interestingly, the proportion of fine roots (Ø < 0.5 mm) and thick root (Ø > 1.0 mm) of Xushu 32 seedlings increased significantly under condition of K deficiency. These results indicate potential genotypic differences in RSA and K absorption ability under K deficiency. Similarly, Wang et al. (2017) under field conditions, indicated that increased K application increased total root length, average root diameter and significantly increased the differentiation from adventitious roots to fibrous roots and tuberous roots. This root traits coupled with added K is beneficial to the early formation of storage roots and number of storage roots per plant, overall root biomass and yield.

However, limited work on the relationship between RSA and NPK can be found for cassava and yams, the most important RTC species in sub-Saharan Africa ^3^. In cassava, Izumi et al. (1999) provided evidence that well-developed branching pattern (i.e., number and length of axile roots and lateral roots) and total root length was associated with water and nutrient absorption and essential for storage root bulking. There has been some follow-up work on the role of cassava RSA and drought tolerance but none for nutrient acquisition (Pardales and Esquibel, 1996; Subere et al., 2009). Adu et al. (2018) corroborated earlier findings and documented root genotypic variation in relative root growth rate, root length, number of nodal roots, root diameter and root branching density in a panel of cassava cultivars bred for high carotenoid content and resistance to cassava mosaic disease (CMD), recommending further studies regarding manipulating cassava RSA for nutrient use efficiency and yield.

Charles-Dominique et al. (2009) conducted an analysis of the tuber monocot, white yam (*Dioscorea cayennensis* subsp. *rotundata* Poir., *Dioscoreaceae*) root system derived from both sexually and vegetatively propagated yams and demonstrated that both seedlings and plants derived from tubers have two distinct root systems that are highly organized. The first type of root system (seminal) is considered transitory (i.e., short-lived) consisting of two root axis categories. The second type of root system (adventitious) is considered permanent and is larger in weight and volume compared to the transitory root system. This adventitious root system is made up of three root axis categories and this is the site for initial tuber formation. Charles-Dominique et al. (2009) concluded the importance of studying the yam root system architecture as a whole and simultaneously in order to understand its growth, development and tuber formation. In a similar study, Hgaza et al. (2012) documented the response of the RSA of water yam (*Dioscorea alata*) and white yam (*Dioscorea cayennensis* subsp. *rotundata*) to mineral fertilizer application under field conditions. Researchers used sequential root coring to assess horizontal and vertical root distribution. Results revealed three root types (seminal, adventitious and tuber roots) and differences in root length density, root mass density and specific root length correlated directly with higher temperature and not with fertilizer application when compared to controls. Hgaza et al. (2012) concluded that tuber formation was independent from seminal and adventitious root development and mineral nutrition did not affect final tuber yield.

The significant resources devoted to the investigation of RSA in cereal crops has led to advances in breeding and selecting RSA for improved NPK acquisition in low-input production environments. Table 2 summarizes the root traits necessary for adaptation to low NPK conditions in rice, maize, and the common bean (*Phaseolus vulgaris* L.) (Lynch and Brown, 1998; Kong et al., 2014). In maize, it has been determined that deeper roots are associated with increased acquisition of N that may leach to lower soil layers (Lynch and Wojciechowski, 2015). In rice, there is evidence that *DEEPER ROOTING 1*, a quantitative trait locus for root growth angle, increased N uptake in N-deficient conditions (Arai-Sanoh et al., 2014). This knowledge has led breeding programs to screen rice and maize genotypes for this desirable trait but also to invest in management practices like nutrient amendments that could improve root growth in rice (Abiven et al., 2015; Ju et al., 2015; Qiao et al., 2018).

**Table 2:** Summary of relevant root traits related to N and P deficiency in rice, maize, and beans.

Species	Nutrient deficiency	Root traits	Reference
Rice	N	Deeper roots	Ogawa et al., 2014; Ju et al., 2015
Maize	N	Low lateral root (LR) branching density, longer LRs	Postma et al., 2014; Yu et al., 2015a; Zhan and Lynch, 2015
		Deeper roots	Lynch and Wojciechowski, 2015; Yu et al., 2015b
		Low crown root number	Saengwilai et al., 2014
Rice	P	Early root growth	Gamuyao et al., 2012
Maize	P	High LR branching density, shorter LRs	Postma et al., 2014
Bean	P	Decreased root metabolic cost, higher root hair length and density	Strock et al., 2018


## Manipulating Root System Architecture for Increased Nutrient Efficiency: Way Forward for Root and Tuber Crops

Advances in manipulating RSA in cereal crops like rice and maize can serve as a model for RTCs. Meaningful advances in rice and maize RSA were made possible by first achieving fundamental understanding of the intrinsic and environmental factors that control RSA. Some of these findings have already been translated into some RTCs, underscoring the importance of translating findings from model crop systems, such as, rice, maize and bean into non-model species, such as, sweetpotato, potato, and yams. Concomitant with the understanding of the biology of RSA, significant investments were made toward the development of minimally intrusive, non-destructive whole-root phenotyping systems (Chen et al., 2011; Kuijken et al., 2015). The development of these phenotyping platforms in turn enabled functional genomics and crop improvement applications (Yang et al., 2013). These phenotyping tools and approaches can be adapted for use in RTCs.

Recent and past advances in understanding RSA have come from the studies on the model plant *Arabidopsis thaliana* and the description of the cellular structure laid the foundation for developmental and genetic work in cereals and other well-studied crops (Dolan et al., 1993; Smith and De Smet, 2012). Similar to RTCs, the *Arabidopsis* root system undergoes secondary thickening under appropriate growth conditions (Dolan and Roberts, 1995; Chaffey et al., 2002). In these and other root crops, root secondary growth followed by starch deposition and increase in root biomass determine the harvestable agronomic yield. This particular area of research has not been extensively studied in RTC under nutrient deficiencies and merits research.

Reduced metabolic cost of soil exploration is important for P capture because continued soil probing is required to increase beyond the depletion of available P in the rhizosphere (Lynch, 2015). For example, the formation of root cortical aerenchyma (RCA) in different crop species is one of the latest advances in our understanding of the impact of nutrient deficiencies in root architecture. RCA is defined as tissue with large intercellular spaces in root cortex normally produced in plant species under hypoxia (Esau, 1977; Drew et al., 1979). However, RCA can be also formed in response to drought and edaphic stresses such as N and S deficiencies (Drew et al., 1989; Bouranis et al., 2003; Fan et al., 2003, Zhu et al., 2010). In maize, genotypes with greater RCA had greater topsoil foraging, P acquisition, growth and yield under low P environments (Galindo-Castañeda et al., 2018). Currently, there are no published studies on the formation of RCA in RTCs.

Another important change in root architecture as a result of nutrient deficiency is the presence or absence of root secondary growth. It has been hypothesized that a decrease in root secondary growth could lessen the carbon cost of producing and sustaining root length to improve the balance between soil exploration use and depletion of growth limiting nutrients (Lynch, 1995). This may be an adaptive strategy to improve the metabolic efficiency of soil foraging under sub-optimal P, where roots will favor primary growth (elongation) over secondary growth (radial swelling) to reach greater probing of soil areas that still hold available P (Lynch, 2007, 2011; Lynch and Brown, 2008; De la Riva, 2010). In bean, secondary root growth under low P is inhibited, but genotypes with higher inhibition of secondary root growth presented reduced root costs, greater P capture, and greater growth under low P environments (Strock et al., 2018). Contrary to the reduced bean secondary root growth model, sweetpotato storage and lateral root growth were not reduced under sub-optimal P levels (Duque and Lynch, 2018).

**FIGURE 1:**
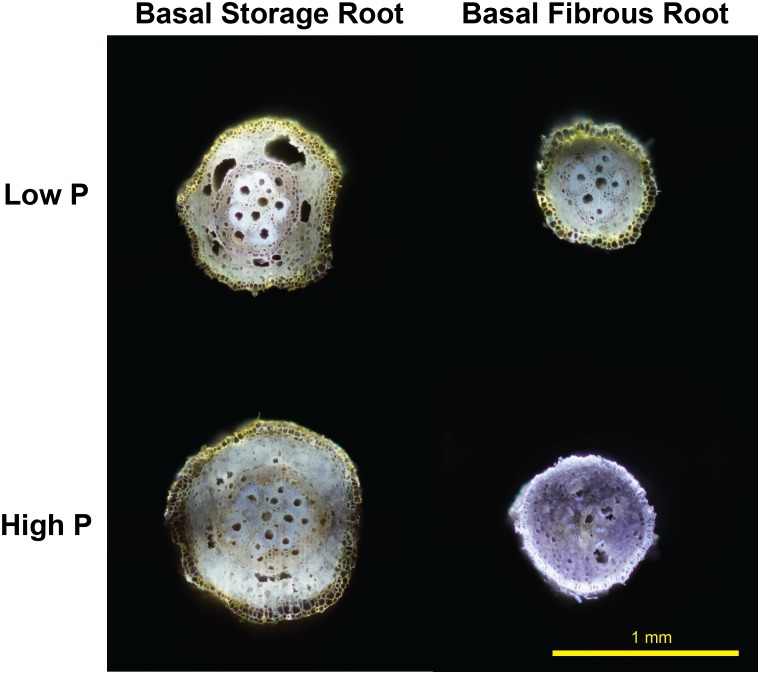
Root cortical aerenchyma-like structure formed after secondary root growth under low phosphorus in sweetpotato storage root and fibrous root at 30 DAP. (Basal meaning root segment farthest from the attachment to the stem node). Root transversal cut sections were performed using laser ablation tomography. Ablation and picture were taken by Peter Ilhardt at Penn State University.

New evidence in sweetpotato RSA under low P environments both in greenhouse and field settings suggests a reduction in metabolic costs of soil exploration with the formation of RCA after root secondary growth in basal cross sections of storage roots but not in lateral roots (Figure 1). These preliminary results suggest a translocation of carbon resources from the storage root to the lateral roots to enhance further soil exploration and/or increase of lateral root branching (Duque and Lynch, 2018). Based on these primary results, RCA merits research on how it can potentially affect final root yield. However, this phenomenon could have profound effects on storage root size, shape and yield, thus future research should focus on the assessment of early versus late bulking genotypes, root genotypic variability and tolerance of sweetpotato under P deficiency, focusing breeding and management efforts for degraded, low input agricultural systems found in Sub-Saharan Africa where sweetpotato as well as other RTCs are staple and subsistence crops.

Indeed, for RTCs in general, species-specific RSA knowledge appears to be at the level of classical morphology and with scant information on the genetic, hormonal, and molecular control of RSA. For root crops in particular, there is a general disconnect between RSA and storage root formation under nutrient deficiencies or water stress, either at the genetic, hormonal and/or molecular level. There has been crop-specific progress on the relationship between RSA and storage root formation in sweetpotato. In particular, it has been shown that lateral root development is a prerequisite to secondary cambium development in the adjacent main root tissue (Villordon et al., 2012). Previously, a cytokinin-regulated *I. batatas* *MADS-box 1* (*IbMADS1*) showed evidence of selective gene expression in meristematic tissue in the stele and in lateral root primordia and it has been proposed that this gene is an integrator at the onset of storage root formation in a network that involves hormones such as jasmonic acid and cytokinin as trigger factors (Ku et al., 2008). *IbMADS1* belongs to the same family as *Arabidopsis nitrate regulated* (*ANR1*), a gene previously shown to be associated with *Arabidopsis* lateral root development in response to nitrate (Zhang and Forde, 1998). In potato, it has been determined that RSA traits such as specific root length of basal roots and total root weight for various root classes are related to final tuber yield (Wishart et al., 2013). Basal roots are important for water uptake and anchorage, whereas stolon roots are connected with nutrient acquisition and tuber formation (Wishart et al., 2013). An earlier work by Sattelmacher et al. (1990) provided evidence that root length and surface area was important for nitrogen acquisition and that a large root system was associated with higher N acquisition.

Despite these efforts, the link between storage root/tuber yield and the carbon partitioning to other root types as well as the regulatory networks involved in RTCs has yet to be established (Khan et al., 2016). However, the cumulative evidence supporting the link between RSA and storage root in sweetpotato and between RSA and tuber yield in potato paves the way forward for more in depth work in sweetpotato and potato as well as similar studies in other RTCs. Root systems are inherently difficult to study and frequently overlooked in research. Due in large part to the RSA work in cereals and other model systems, novel tools and approaches have been developed to non-invasively measure root development in laboratory, greenhouse and field settings. Traditional methods for studying root systems include rhizotrons (Nagel et al., 2012), rhizoboxes (Lemming et al., 2016), and excavation (Bucksch et al., 2014). Image-based systems have been developed to overcome the phenotyping bottleneck, including X-ray computed tomography (Mairhofer et al., 2012), magnetic resonance imaging (Metzner et al., 2015; van Dusschoten et al., 2016), and ground penetrating radar (Guo et al., 2013), among others. However, wide scale adoption of some of these methods continued to be hampered by prohibitive costs and lack of accessibility. In RTCs, some of the non-destructive methods that have been used in the field with limited samples include rhizotrons or rhizotron-like methods in cassava (Tscherning et al., 1995), potato (Parker et al., 1991) and sweetpotato (Villordon et al., 2011). The use of viewing devices like minirhizotrons have actually been shown to interfere with storage root formation (Villordon et al., 2011), further limiting the options to study the relationship between RSA and storage root formation in root crops. Taken together, it appears that critical barriers to progress in understanding crop-specific RSA attributes in RTCs include the lack of a model system for interpreting the relationship between RSA and storage root and tuber yield and the current prohibitive costs of non-destructive, high-throughput image-based phenotyping tools.

One way forward to overcome these barriers is to use the sweetpotato (dicot, storage root), cassava (dicot, storage root), potato (dicot, tuber) and yam (monocot, tuber) as primary model systems for understanding the connection between RSA and agronomic yield in RTCs, respectively. These RTCs are considered the most important calorie-producing staple crops for smallholder subsistence farmers combined with low input agriculture on marginal lands typically located in underdeveloped countries.

Strategic translational research using data on RSA and NPK uptake from *Arabidopsis* and cereal model systems should continue using key RTC cultivars, as a means to rapidly validate key findings. Once validated, information on key RSA traits should be immediately forwarded to breeding programs for further studies and validation in breeding populations. These breeding programs should take advantage of available resources for adapting phenotyping methods for integrating root traits into existing breeding objectives. Finally, international agricultural research centers, as well as national institutions that have mandates in RTCs, should continue to intensify RSA research investments into their current and future research priorities, especially under the threat of climate change, vulnerable agro-ecological landscapes and poverty. During the first Green Revolution, improved rice and wheat varieties were rapidly adopted in tropical and subtropical regions that had good irrigation systems or reliable rainfall (Evenson and Gollin, 2003). The spread of these improved varieties was associated with the activity of international agricultural research centers (Evenson and Gollin, 2003). It has been suggested that a second Green Revolution, one that incorporates RSA traits, is vital to improve the yield of crops grown in infertile soils by farmers with little or no access to fertilizers (Lynch, 2007). Just like the first Green Revolution, such research centers will likely have an important role in ushering in the second Green Revolution (Zeigler and Mohanty, 2010).

## Conclusion

The agronomic significance of understanding the regulation of RSA development is now widely accepted because of its role in soil resource acquisition under edaphic stress. In well-studied “model” crop species like rice, maize, and soybeans, the knowledge of RSA has already led to measurable gains in the ability of these crops to exploit soil resources under low-input conditions. For example, Zhao et al. (2004) reviewed by Li et al. (2016), showed that an applied core collection of soybeans with shallow root architecture presented improved spatial root aggregations enhancing P explorations resulting in higher P efficiency and yield. In maize, Liu et al. (2018) showed a positive and significant correlation between grain yield and both total root number and total root length. The tools and approaches that have been used in cereals can be applied to RTCs, potentially reducing the costs of research and development, however, these novel tools and approaches have to be sufficiently modified to account for real-time tuber and storage root development and growth as no single phenotyping platform nor specialized analytical software exists at the moment for RTCs. Unraveling the role of RSA in RTC nutrient uptake will improve global food security, especially in regions with marginal soil fertility and low-input agricultural conditions.

## Data Availability

All datasets generated for this study are included in the manuscript and/or the supplementary files.

## Author Contributions

LD and AV conceptualized and wrote the review.

## Conflict of Interest Statement

The authors declare that the research was conducted in the absence of any commercial or financial relationships that could be construed as a potential conflict of interest.

## Footnotes

1 http://www.fao.org/docrep/x5415e/x5415e01.htm

2 http://www.fao.org/docrep/005/y9422e/y9422e0d.htm

3 www.fao.org/docrep/x5415e/x5415e01.htm
